# Strategy to Estimate Sample Sizes to Justify the Association between MMP1 SNP and Osteoarthritis

**DOI:** 10.3390/genes13061084

**Published:** 2022-06-17

**Authors:** Chung-Cheng Kao, Hsiang-En Hsu, Jen-Chieh Lai, Hsiang-Cheng Chen, Su-Wen Chuang, Meng-Chang Lee

**Affiliations:** 1 Tri-Service General Hospital Songshan Branch, National Defense Medical Center, Taipei 10581, Taiwan; kao8267kq@gmail.com; 2 School of Public Health, National Defense Medical Center, Taipei 11490, Taiwan; eve998877@gmail.com (H.-E.H.); suwen7@gmail.com (S.-W.C.); 3 Orthopaedic Department, Armed Forces General Hospital, Taichung 41152, Taiwan; shoulia2001@yahoo.com.tw; 4 School of Medicine, National Defense Medical Center, Taipei 11490, Taiwan; 5 Division of Rheumatology/Immunology/Allergy, Department of Internal Medicine, Tri-Service General Hospital, National Defense Medical Center, Taipei 11490, Taiwan; hccheng@mail.ndmctsgh.edu.tw; 6 Graduate Institute of Life Sciences, National Defense Medical Center, Taipei 11490, Taiwan

**Keywords:** osteoarthritis (OA), *MMP1*, Meta-analysis, Trial sequential analysis (TSA)

## Abstract

Background: the impact of knee osteoarthritis (OA) poses a formidable challenge to older adults. Studies have reported that genetic factors, such as *MMP1*, are one of important risk factors for knee OA. Although the relationship between the genetic polymorphism of *MMP1* rs1799750 and the risk of knee OA has been explored, conclusions have been nonunanimous and pending due to research sample sizes, one of determinants in studying genetic polymorphisms associated with disease. Objective: to establish a model to assess whether the genetic polymorphism of *MMP1* rs1799750 is associated with knee OA based on an estimation of sample sizes. Methods: samples were collected from a case–control and meta-analysis study. In the case–control study, patients who underwent knee X-ray examinations based on the Kellgren–Lawrence Grading System (KL) as diagnostic criteria were recruited at the Health Examination Center of the Tri-Service General Hospital from 2015 to 2019. Gene sequencing was conducted using iPLEX Gold. Those with unsuccessful gene sequencing were excluded. Finally, there were 569 patients in the knee OA group (KL ≥ 2) and 534 participants in the control group (KL < 2). In the meta-analysis, we used the databases PubMed, EMBASE, and Cochrane to search for studies on the relationship between *MMP1* rs1799750 and knee OA. Next, we adopted the trial sequential analysis (TSA) method to assess whether sample sizes were sufficient or not to determine the risk of the genetic polymorphism of *MMP1* rs1799750 on knee OA in Caucasians and Asians. Results: in Caucasians, the *MMP1* rs1799750 was not significantly associated with knee OA with an odds ratios (OR) of 1.10 (95% confidence interval, CI: 0.45–2.68). Some extra 8559 samples were needed to conclude this relationship in Caucasians by the TSA model. In Asians, neither our case–control study results (*n* = 1103) nor a combination of samples from the case–control and meta-analysis results showed an association between *MMP1* rs1799750 and knee OA. The OR (95% CI) was 1.10 (0.81–1.49) in a combination of Asian samples. Some extra 5517 samples were needed to justify this relationship in Asians by the TSA model. Conclusions: this research shows that an extra 8559 and 5517 samples are needed in Caucasians and Asians, respectively, in order to justify the association between *MMP1* rs1799750 and knee OA.

## 1. Introduction

Osteoarthritis (OA) is the most common joint disease and the main cause of disability in older adults globally [1]. Among all factors in OA, the genetic factor is particularly important, and the heritability of knee OA is approximately 45% [2]. Studies have reported that OA is primarily affected by genetic risk factors because of common population polymorphisms in multiple genes [3]. More research is needed to identify more candidate genes and evaluate their effects.

*MMP1*, one of the matrix metalloproteinases (MMPs), which is located on the long arm of chromosome 11 (11q.23), plays an important role in collagen degradation [4]. Articular cartilage comprises the chondrocytes and extracellular matrix (ECM). MMP1 is able to degrade type I, II, and III collagen in ECM. Overexpression of *MMP1* in chondrocytes stimulates the degradation of cartilage collagen and proteoglycan, leading to pathological cartilage damage and resulting in OA [5,6]. Among the single-nucleotide polymorphisms (SNP) in *MMP1* promoter, rs1799750, a type of a guanine insertion polymorphism (2G polymorphism) at position −1607 and together with an adjacent adenosine forming the sequence 5′-GGA-3′, comprising an ETS transcription factor family DNA-binding site, results in enhancing transcriptional activity when compared to that of a guanine deletion polymorphism (1G polymorphism) [7,8]. Patients with OA feature a higher expression of MMP in the cartilage than that of normal, of which MMP is positively associated with the expression of cytokines, such as interleukin-1beta (IL-1beta) and tumor necrosis factor-α (*TNF*-α) [9]. 

Five chosen studies (please refer to Meta-analysis in Materials and Methods) have investigated the association between the rs1799750 polymorphism and knee OA until now [10,11,12,13,14]. However, conclusions have been nonunanimous and pending. Two studies report that the minor allele (2G allele) carriers have higher risk of knee OA [11,14]; another study proposes that the minor allele (2G allele) carriers have a lower risk of knee OA [10]; the others show null association between the *MMP1* rs1799750 polymorphism and knee OA [12,13].

To confirm the relationship between *MMP1* rs1799750 and knee OA, we considered that the sample size is one of determinants in greater reliable meta-analysis [15]. The total participants in Asians and Caucasians were 1121 and 731, respectively [10,11,12,13,14]. Too few participants may disturb the credibility of results. Thus, the aim of this study was to examine whether those sample sizes were sufficient or not to judge those inconsistent results. To fulfill this task, we adopted the trial sequential analysis (TSA), which not only provided estimation of the sample sizes’ function but also tested the relationship between *MMP1* rs1799750 and knee OA [16,17]. Besides, to confirm previous research, we also conducted a case–control study to validate the association of *MMP1* rs1799750 polymorphisms and knee OA in Taiwan.

## 2. Materials and Methods

### 2.1. Case–Control Study

#### 2.1.1. Ethical Issues

This research was approved by the institutional review board (TSGH-2-102-05-028) of the Tri Service General Hospital (TSGH). Volunteers signed the consent form after the investigators had provided an explanation of the study.

#### 2.1.2. Subjects

A total of 1103 participants (569 case and 534 Control) comprising controls and patients with ages of ≥65 years old were recruited in this study. All participants used Taipei City senior medical check-ups between January 2015 and December 2019 at the TSGH, a medical teaching hospital at the National Defense Medical Center, Taipei, Taiwan. The check-up is a government welfare program for older adults who have been registered as Taipei City residents for more than 1 year.

The patients’ information was confirmed when participants underwent check-ups. Participants who had received study information, understood the process, and provided written consent were enrolled. We excluded participants who unable to draw enough blood samples. Exclusion criteria were the participants without knee X-ray data, other etiologies of knee joint disease, such as inflammatory arthritis, posttraumatic or postseptic arthritis, skeletal or developmental dysplasia, and unsuccessful genetic sequencing results. A total of 92 samples could not be genotyped by our genotyping process; therefore, 1103 (92.3%) samples were included in the genetic analyses.

All participants had a radiographic examination of both knees with anterior–posterior and lateral views. Besides, weight-bearing and foot-map positioning were recorded. Knee radiographs were read and scored by an orthopedic surgeon using the Kellgren–Lawrence (KL) grading system [18]. In the KL system, the stages range from 0 to 4 grades. For patients with different KL grades in each knee, the more advanced grade was used for evaluation. Knee OA was defined as KL grades of ≥2. Finally, this study included 569 knee OA patients and 534 healthy controls.

#### 2.1.3. Genomic DNA Extraction and Genotyping

Approximately 10 mL peripheral blood was intravenously extracted from participants by a physician or nurse. Genomic DNA was isolated using standard procedures, such as phenol/chloroform methods. The rs1799750 SNP was genotyped using the Mass array iPLEX Gold SNP genotyping method. Genotyping was performed under blind conditions. To validate results, at least 10% of samples were randomly selected for repeated genotyping, and the reproducibility rates were 98.1%.

#### 2.1.4. Statistical Analysis

Continuous variables of the general demographic data are expressed as the mean and standard deviation (mean ± SD) and were tested using a t-test. Differences in genotype and allele frequencies between knee OA patients and healthy controls were tested using the Chi-squared test. Odd ratios (ORs) and 95% confidence intervals (CIs) for the risk of knee OA were calculated using logistic regression. Calculation of genetic polymorphisms and knee OA risk was expressed using allele type, genotype, and dominant/recessive models. A *p* value of less than 0.05 was considered significant. R software version 3.4.4 (Vienna, Austria) was used for statistical analyses.

### 2.2. Meta-Analysis

#### 2.2.1. Search Methods and Criteria for Study Consideration

The PRISMA and Meta-analysis on Genetic Association Studies checklists are provided in Supplementary Table S1 [19]. Research about the correlations between *MMP1* rs1799750 and the risk of OA was searched in PubMed, EMBASE, and Cochrane using “*MMP1* rs1799750” and “Osteoarthritis” and its synonyms as the search keywords up to 19 November 2020 (Supplementary Table S2), and only studies written in English were targeted. In addition, our research team manually reviewed all meta-analysis studies. The inclusion criteria for the studies to be enrolled were as follows: (1) case–control or cross-sectional studies, (2) studies using the KL classification as the diagnostic criteria, such as the case (KL ≥ 2) and control groups (KL < 2), and (3) studies that included subjects aged > 18 years old.

#### 2.2.2. Data Extraction

Two reviewers worked independently to collect literature data, including the first author’s surname, year of publication, country where the research was conducted, ethnicity of the research group, and gene distribution of the case and control groups. All chosen papers were assessed using the Newcastle–Ottawa Scale, and all received scores of >6 points.

#### 2.2.3. Statistical Analysis

Each chosen article was described using an appropriate ratio or average value. In the meta-analysis, ORs with 95% CIs were used to explore the correlation between *MMP1* rs1799750 and OA. The I^2^ test was used to evaluate heterogeneity, where I^2^ of >50% indicated moderate to high heterogeneity. Egger’s regression and funnel plot were used to examine the symmetry after incorporation of the two parts. Further, genetic models including allele, dominant, and recessive models were used to calculate the risk level of *MMP1* rs1799750 for OA by combining the calculation results via a random-effects model. The significance level was set at 0.05, and the packages “metafor” [20] and “meta” [21] of R software version 3.3.1 (Vienna, Austria) were used. The TSA method was used to estimate sample sizes and to verify the results of the meta-analysis [16]. Stratification analysis was conducted by race, comprising Caucasians and Asians. Type I error was set at 0.05; power was set at 0.8; heterogeneities in Caucasians and Asians were set at 94 and 80%, respectively. The literature review showed that the OR of correlation between MMP1 rs1799750 and OA was approximately 1.3. In the present study, considering that the 2G allele was a potential risk factor, the OR value was set at 1.3. The Taiwan Biobank and 1000 Genome databases were used as references for minor allele frequency, which were 0.47 and 0.47 for Caucasians and Asians, respectively.

## 3. Results

### 3.1. Case–Control Study

Table 1 showed the baseline demographic characteristics of the case–control population. Of 1103 subjects, there were 534 participants in the control group with mean ± SD of 71.60 ± 6.86 years old (263 men and 271 women) and there were 569, patients in the case group with a mean ± SD of 73.55 ± 7.27 years old (204 men and 365 women). The number of men in the case group was lower than that in the control group (*p* < 0.001), and the average age of the case group was higher than that of the control group (*p* < 0.001). Neither the distribution of the 2G allele between the case and control groups (*p* = 0.762) nor the comparison of 2G allele to 1G allele with an adjustment of covariates in association with OA (OR: 0.96, 95% CI: 0.75–1.24) showed a significant association (Table 2). To further verify the results, dominant and recessive models were examined, wherein neither of two models was significant. Therefore, *MMP1* rs1799750 did not have a significant association with knee OA in the case–control study.

### 3.2. Meta-Analysis

**Figure 1:**
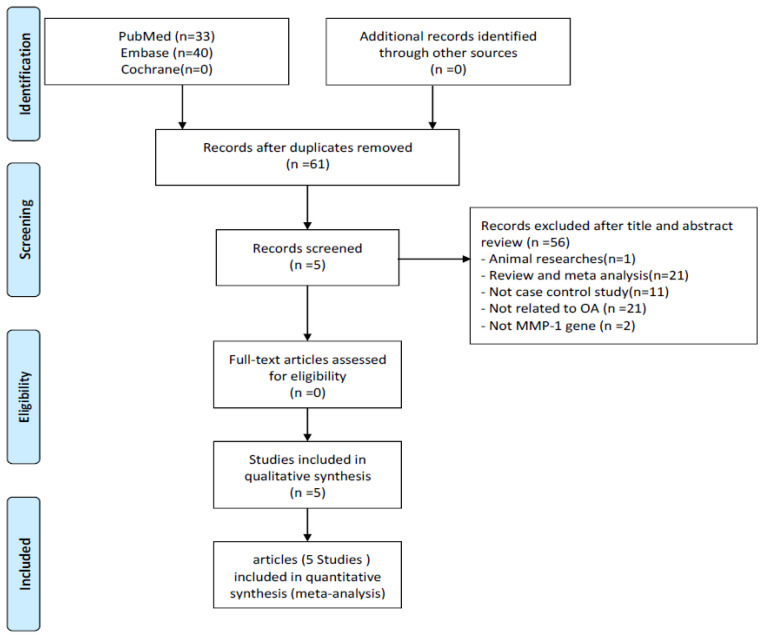
Flow diagram of the identification process for eligible studies.

The flow chart of the literature review is shown in Figure 1. In the meta-analysis, 33 articles were collected from PubMed. Then, another 40 articles were collected by manual review from EMBASE, reaching the total number of articles to 73. After removing duplicate articles, a total of 61 articles were included in the inclusion screening, which was performed based on the titles and abstracts. Among these, 21 articles were excluded because they were reviews or meta-analysis and the other 35 articles were irrelevant. Finally, five articles were included for analysis. The basic descriptions of the articles included in the meta-analysis are shown in Supplementary Table S3; the quality evaluation are shown in Supplementary Table S4.

**Figure 2:**
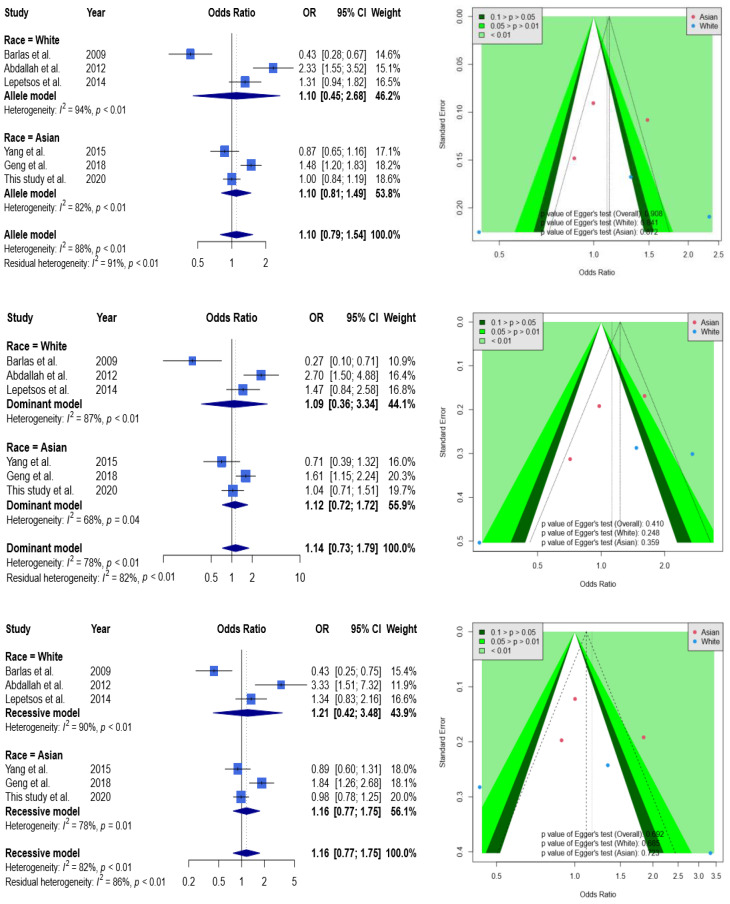
Forest plot and funnel plot regarding the association between MMP1 rs1799750 and knee OA. Selected results from the meta-analysis of MMP1 rs1799750 and knee OA. The top left subplot is a forest plot based on an allele model assumption (reference: 1G allele) and the top right subplot is a funnel plot based on the allele model assumption. The results obtained with the dominant (1G1G + 1G2G vs. 2G2G) and recessive (1G1G vs. 2G2G + 1G2G) models are presented at the middle and bottom. All results are nonsignificant.

According to the Figure 2, none of results were significant in allele (allele 2G to allele 1G), dominant (1G1G + 1G2G vs. 2G2G), and recessive (1G1G vs. 2G2G + 1G2G) models. For example, OR was at 1.10 with a 95% CI from 0.79 to 1.54 in the allele model. When stratified by race, ORs were at 1.10 with a 95% CI from 0.45 to 2.68 and at 1.10 with a 95% CI from 0.81 to 1.49 in Caucasians and Asians, respectively. A funnel plot was used to demonstrate the association between ORs and standard error in the allele model, with each point representing a study. No significant asymmetry was discovered between the articles. Likewise, none of results from dominant and recessive models showed significance.

### 3.3. TSA Evaluation

**Figure 3:**
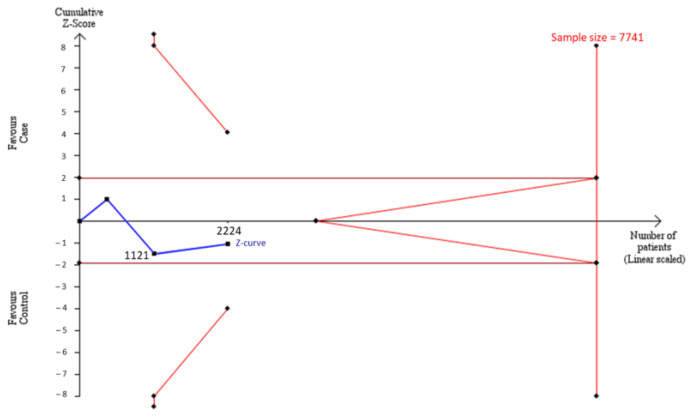
Trial Sequential Analysis (TSA) in Asians. We performed a TSA using an allele model assumption but replaced the allele count with the sample size (divided by 2). Detailed settings: Significance level = 0.05; Power = 0.8; ratio of controls to cases = 1; hypothetical proportion of controls with 2G allele = 0.47; least extreme OR to be detected = 1.3; I^2^ (heterogeneity) = 88%.

**Figure 4:**
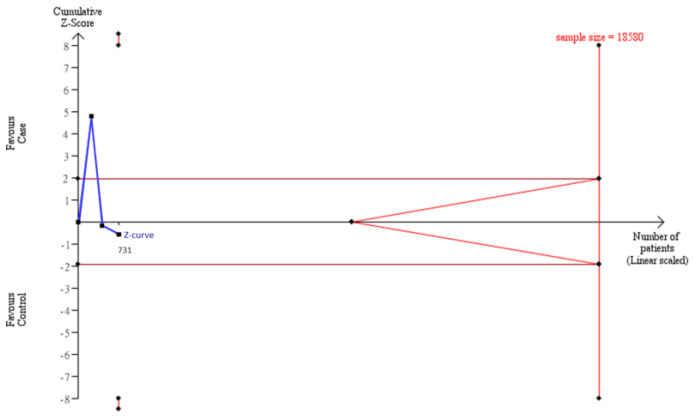
Trial Sequential Analysis (TSA) in Caucasians. We performed a TSA using an allele model assumption but replaced the allele count with the sample size (divided by 2). Detailed settings: Significance level = 0.05; Power = 0.8; ratio of controls to cases = 1; hypothetical proportion of controls with 2G allele = 0.47; least extreme OR to be detected = 1.3; I^2^ (heterogeneity) = 80%.

To estimate whether sample sizes were satisfied with the requirement of a decisive conclusion, we used a TSA model to examine a combination of our case–control samples (*n* = 1103) and samples from meta-analysis (*n* = 1121) in Asians. The data showed that the total of 2224 participants was not adequate to meet the requirement of sample sizes to judge a decisive conclusion, and some extra 5517 samples were needed to justify this relationship in Asians by the TSA model (Figure 3). Similarly, the cumulative Caucasian samples (*n* = 731) also did not satisfy the requirement of sample sizes to judge a decisive conclusion, and some extra 8559 samples were needed to justify this relationship in Caucasians by the TSA model (Figure 4.)

## 4. Discussion

The results of the present study showed that the gene polymorphism of *MMP1* rs1799750 was not significantly associated with knee OA, which is inconsistent with some previous studies. When compared with the previous studies in Caucasians, Barlas et al. reports that, considering the 1G allele as the benchmark, the 2G allele indicated a 0.43 times chance of risk of knee OA, thus concluding that the 2G allele is an apparently protective factor [10]. However, these results might be attributed to a relatively small sample size. Similarly, in the study by Abdallah et al., considering the 1G allele as the benchmark, the 2G allele indicated a 2.33 times chance of risk of OA, thus concluding that the 2G allele is an apparent risk factor [11]. Leptsos et al. proposes that the 2G allele is not significantly associated with OA [12]. Consistent with the results of Yang et al.’s study in Asians, the genetic polymorphism of *MMP1* rs1799750 is not significantly associated with OA [13]. However, Geng et al. point out that the genetic polymorphism of *MMP1* rs1799750 is significantly associated with OA [14].

In the meta-analysis part of the present study, it was found that *MMP1* rs1799750 was not associated with OA. In the meta-analysis by Xu et al. in 2019, in which five articles were included and one of these was about temporomandibular joint arthritis, the gene polymorphism of *MMP1* rs1799750 was not significantly associated with OA [22]. Likewise, in studies by Peng et al. in 2019 [23] and by Liu et al. in 2020 [24], in which six or seven articles were included, neither of them shows an association between the gene polymorphism of *MMP1* rs1799750 and OA.

In the present study, a subgroup analysis stratified by age was conducted (Supplementary Figure S1). In the allele model of *MMP1* rs1779750, the heterogeneity of the group aged > 60 years was found to be slightly reduced, yet the polymorphism of *MMP1* rs1779750 was not associated with knee OA. Meanwhile, in the group aged < 60 years old, it showed that *MMP1* rs1779750 was associated with knee OA (OR: 1.80, 95% CI: 1.16–2.78). It is still unclear why the association between *MMP1* rs1799750 and knee OA only exists in younger individuals. Presumably, aging itself, the pathogenic factors, and pathogenesis in older adults compounds disease and thus disperses the contribution from genetic effects. In addition, the differences in lifestyle and environmental factors among different groups of people are related to the occurrence of OA and may also interact with genes [22].

The present study has three strengths. (1) Estimation of the sample size by TSA: In the past, meta-analyses rarely involved a method to estimate whether their sample sizes were adequate or not to reach the benchmark for a definite conclusion. In the present study, the TSA method suggested the sample sizes were insufficient and provided how many exact numbers of samples were needed in both studies of Caucasians and Asians. (2) In general, epidemiological studies only use their own samples for analysis, while meta-analyses only analyze data from published articles. The present study combined the methods used in traditional observational research and meta-analysis techniques to further increase the sample size to improve the power of evidence. Then, TSA was introduced to examine the sample sizes that confirmed the relationship between *MMP1* rs1799750 and knee OA in the Asian population. (3) In the present study, the results of the two parts were incorporated using a random-effects model, which can avoid serious errors that may be caused by a model’s selection based on the level of heterogeneity [25].

However, the present study has two limitations. First, only English articles were included, and those published in other languages were not included in the meta-analysis, which might lead to bias. Second, the high heterogeneity could not be explained, which might imply potential gene-gene and gene-environment interactions. Our previous study developed a revised version of meta regression, known as case-weighted meta regression, to analyze the gene-gene and gene-environment interactions using average population information [26]. We suggest that further researchers should provide complete population characteristics for future meta-analysis.

## 5. Conclusions

We found that *MMP1* rs1799750 was not associated with the risk of knee OA in a case–control or a combination of case–control and meta-analysis study. However, we used a TSA model to examine the cumulative number of samples and found that an extra 8559 and 5517 samples are needed in Caucasians and Asians, respectively, in order to justify the association between the *MMP1* polymorphism and OA. Thus, we believe that this method can provide scientists the means to estimate whether their sample sizes are adequate or not to conclude their discoveries in disease and gene association studies. In the future, the effects of gene–environmental factors on the risk of knee OA shall be taken into account.

## Figures and Tables

**Table 1:** Baseline demographic characteristics of the study subjects.

	Knee OA Group (*n* = 569)	Control Group (*n* = 534)	*p* Value
Gender (%)			<0.001 *
Male	204 (35.9%)	263 (49.3%)	
Female	365 (64.1%)	271 (50.7%)	
Age (mean ± SD)	73.55 ± 7.27	71.60 ± 6.86	<0.001 *
BMI (mean ± SD)	24.63 ± 3.61	24.12 ± 3.33	0.018 *
25th percentiles	22.33	21.78	
Median	24.38	23.92	
75th percentiles	26.73	25.88	
KL Grade (%)			<0.001 *
0	0	22 (4.1%)	
1	0	512 (95.9%)	
2	420 (73.8%)	0	
3	79 (13.9%)	0	
4	70 (12.3%)	0	

Knee OA group: KL ≥ 2; Control group: KL < 2. Body mass index (BMI). * *p* value < 0.05.

**Table 2:** Association between genetic polymorphism of *MMP1* rs1799750 and knee OA.

	Knee OA Group (*n* = 569)	Control Group (*n* = 534)	Crude-OR (95% CI)	*p* Value	Adj-OR ^a^ (95% CI)	*p* Value
Genotype						
1G1G	63(11.1%)	61(11.4%)	1.00		1.00	
1G2G	262(46.0%)	242(45.3%)	1.05 (0.71–1.55)	0.814	1.07 (0.71–1.63)	0.734
2G2G	244(42.9%)	231(43.3%)	1.02 (0.69–1.52)	0.911	1.02 (0.67–1.55)	0.930
Allele Model						
1G	388(34.1%)	364(34.1%)	1.00		1.00	
2G	750(65.9%)	704(65.9%)	1.00 (0.84–1.19)	0.995	0.96 (0.75–1.24)	0.762
Dominant Model						
1G1G	63(11.1%)	61(11.4%)	1.00		1.00	
1G2G + 2G2G	506(88.9%)	473(88.6%)	1.04 (0.71–1.51)	0.854	1.05 (0.71–1.55)	0.818
Recessive Model						
1G1G + 1G2G	325(57.1%)	303(56.7%)	1.00		1.00	
2G2G	244(42.9%)	231(43.3%)	0.98 (0.78–1.25)	0.900	0.96 (0.75–1.24)	0.762

Knee OA group: KL ≥ 2, Control group: KL < 2. ^a^: adjustment covariates with age, gender, and BMI. Minor allele frequency (MAF): Taiwan Biobank: 47%; 1000 Genome: 47%.

## Data Availability

Not available.

## Supplementary Materials

The following supporting information can be downloaded at: www.mdpi.com/article/10.3390/genes13061084/s1. Supplementary Table S1. PRISMA 2009 Checklist. Supplementary Table S2. Search strategies and detailed records. Supplementary Table S3. Basic description of articles included in the meta-analysis. Supplementary Table S4. Quality evaluation of articles included in the meta-analysis. Supplementary Figure S1 Forest plot of the age-stratified analysis.
